# Novel Magnetic Resonance Imaging Tools for the Diagnosis of Degenerative Disc Disease: A Narrative Review

**DOI:** 10.3390/diagnostics12020420

**Published:** 2022-02-06

**Authors:** Carlo A. Mallio, Gianluca Vadalà, Fabrizio Russo, Caterina Bernetti, Luca Ambrosio, Bruno Beomonte Zobel, Carlo C. Quattrocchi, Rocco Papalia, Vincenzo Denaro

**Affiliations:** 1 Unit of Diagnostic Imaging and Interventional Radiology, Campus Bio-Medico University of Rome, 00128 Rome, Italy; c.mallio@unicampus.it (C.A.M.); c.bernetti@unicampus.it (C.B.); b.zobel@unicampus.it (B.B.Z.); c.quattrocchi@unicampus.it (C.C.Q.); 2 Department of Orthopaedic and Trauma Surgery, Campus Bio-Medico University of Rome, 00128 Rome, Italy; g.vadala@unicampus.it (G.V.); l.ambrosio@unicampus.it (L.A.); r.papalia@unicampus.it (R.P.); denaro@unicampus.it (V.D.)

**Keywords:** low back pain, intervertebral disc, intervertebral disc degeneration, magnetic resonance imaging, T2 mapping, spectroscopy, spine imaging

## Abstract

Low back pain (LBP) is one of the leading causes of disability worldwide, with a significant socioeconomic burden on healthcare systems. It is mainly caused by degenerative disc disease (DDD), a progressive, chronic, and age-related process. With its capacity to accurately characterize intervertebral disc (IVD) and spinal morphology, magnetic resonance imaging (MRI) has been established as one of the most valuable tools in diagnosing DDD. However, existing technology cannot detect subtle changes in IVD tissue composition and cell metabolism. In this review, we summarized the state of the art regarding innovative quantitative MRI modalities that have shown the capacity to discriminate and quantify changes in matrix composition and integrity, as well as biomechanical changes in the early stages of DDD. Validation and implementation of this new technology in the clinical setting will allow for an early diagnosis of DDD and ideally guide conservative and regenerative treatments that may prevent the progression of the degenerative process rather than intervene at the latest stages of the disease.

## 1. Introduction

Low back pain (LBP) is one of the leading causes of disability worldwide, with a peak prevalence in adult and elderly patients. It represents a severe economic and social burden on healthcare systems [1]. Axial spinal pain and neuralgia can arise from degenerative conditions of the spine, including facet joint syndrome, sacroiliac joint osteoarthritis, and degenerative disc disease (DDD).

The intervertebral disc (IVD) shows a peculiar architecture tailored to its biomechanical tasks. Healthy IVDs are between the cartilaginous endplates (CEPs) of two adjacent vertebrae bodies. They are composed of (1) the annulus fibrosus (AF), which is a peripheral structure formed by 15–40 dense parallel concentric lamellae rich in type I collagen that provide resistance against tensile forces [2,3], and (2) the nucleus pulposus (NP), which is the central part of the IVD, enveloped in the AF, providing loading and compressive resistance. Compared with the AF, the NP is a less structured gelatinous substance containing randomly distributed type II collagen fibrils in a highly hydrated extra-fibrillar matrix rich in proteoglycans (especially aggrecan), which provides the NP with its water-binding properties [2]. Hence, the proteoglycan and water contents are maximal in the NP, while collagen content is more prevalent and organized in the AF [3,4]. 

IVDs are prone to degenerative changes, whose prevalence is strongly associated with mechanical overload, overuse, aging, high body mass index (BMI), diabetes, smoking, and genetic factors [5,6,7,8]. At the initial stage of DDD, due to increased catabolism and the decreased production of extracellular matrix components, glycosaminoglycan (GAG) depletion occurs in the NP. Subsequently, proteoglycan’s (PG’s) water-binding capacity reduces, with a loss of fluid pressurization and impairment of mechanical function, together with changes in collagen orientation and abnormal compressive force transmission to the AF, eventually leading to AF lamellar disruption [2,9,10]. In more advanced stages, other changes in disc material characteristics with abnormalities of AF tensile properties occur, leading to structural damage, such as disc height reduction, annular tears, rim lesions, and osteophyte formation [9,11]. The metabolic imbalance of NP involves decreased water content, progressive loss of proteoglycan, and damage to the collagen matrix [12].

DDD usually presents with mechanical discogenic LBP, which worsens with forward flexion and axial loading while being relieved in supine position. However, DDD sequelae, such as disc herniation, degenerative spondylolisthesis, and spinal stenosis, may lead to neural compression and further aggravate the symptoms, with the onset of radicular pain and neurogenic claudication. However, it is important to exclude the causes of non-discogenic LBP, including abdominal aortic aneurysm, pneumonia, nephrolithiasis, infections, gynecological disorders, cancers, and thrombosis of the inferior vena cava [13,14]. Routine standing anterior–posterior and lateral–lateral X-ray is usually the first imaging method to investigate DDD. Typical signs include disc height reduction, osteophyte formation, facet hypertrophy, and alterations in segmental alignment [15].

Magnetic resonance imaging (MRI) is the most widely used technique for specifically assessing the IVD in healthy and degenerative conditions. Based on proton density, water content, and biochemical composition, MRI depicts disc hydration and morphological features [16]. Apart from the advantage of being radiation-free, it is characterized by an excellent soft-tissue contrast and the possibility to perform a multiplanar and multiparametric evaluation of spinal tissues. Therefore, MRI has been established as the standard imaging modality to detect IVD diseases. However, current protocols assess DDD based on gross morphological changes, which are graded using discontinuous and somewhat subjective scales that do not address the underlying, complex pathophysiological processes [17]. In the last decade, innovative MRI techniques have been investigating diverse parameters, including relaxation times, magnetization transfer (MT), spectroscopy, and apparent diffusion coefficient (ADC), to analyze the biochemical composition of the IVD at different stages of DDD.

In this review, we summarized the state of the art regarding the latest MRI technology for diagnosing DDD, describing in detail the novel methodologies and tools that have been under development to detect DDD at the earliest stages.

## 2. Intervertebral Disc: Anatomy and Biomechanics

Each tissue composing the IVD, namely, the NP, AF, and CEPs, presents structural characteristics that are intimately devoted to its biological and biomechanical properties.

The primary role of the NP is to support mechanical load via osmotic and hydrostatic pressure. A high negatively charged proteoglycan content establishes an environment rich in Na^+^ ions and, consequently, water, which explains the high swelling pressure and imbibition of the NP in physiological conditions [18]. However, NP osmotic pressure (and hence, water content) is not steady but recurrently changes based on mechanical load; indeed, it decreases by 20–25% during physical activity and it is restored at rest, with values ranging between 430 and 500 mOsm/L in vivo [19]. Due to the significant hydration and gelatinous consistency, the NP has been considered a viscoelastic tissue with both fluid and solid properties [20]. Under low deformation, the NP shows a constant permeability and a linear relationship between stresses and strains. Following moderate and high deformations, such parameters change in a strain-dependent fashion [21].

The AF has the primary function of constraining the NP, supporting vertical loads, and restraining excessive motion between vertebral bodies. It is mainly composed of collagen, with a progressive decrease in the collagen I/collagen II ratio and proteoglycans from the outer AF to the inner AF [22]. This topology is associated with a change in the loading environment from more tension in the outer AF to more compression toward the inner AF. In addition, the collagenous component of the AF provides the tissue with nonlinear properties. More specifically, the collagen fibrils exhibit a low stiffness at low strains as they progressively uncrimp. Once the fibers are completely stretched, any strain increase produces a linear increment in fiber stress until reaching the breakage point [23]. Furthermore, the specific fiber orientation and angle between concentric lamellae (~60°) provide the AF itself with anisotropic properties (i.e., the capacity to exhibit direction-dependent mechanical properties) [24].

The CEPs form the interface between the vertebral bodies, AF, and NP. Like hyaline cartilage, it is mainly composed of collagen II and proteoglycans, with high water content. The CEPs are provided with high hydraulic permeability, suggesting their role to guarantee the transport of fluids, nutrients, and waste products to the cells in the NP and inner AF [25].

## 3. Magnetic Resonance Imaging in Intervertebral Disc Degeneration

**Figure 1:**
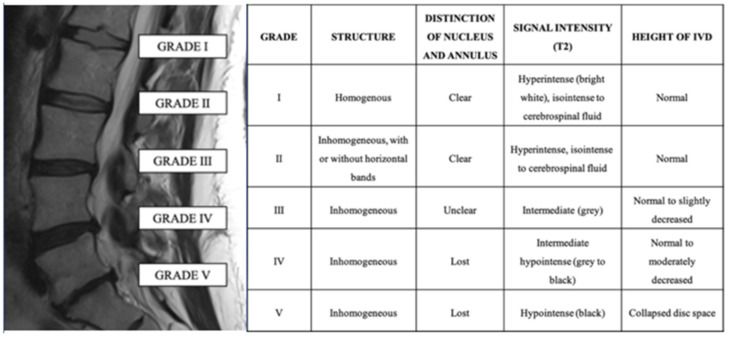
Sagittal T2 image of the lumbar spine (left panel) and explanation of the Pfirmann grading system to assess disc degeneration.

MRI plays a pivotal role in LBP diagnosis. It is considered the best non-invasive method to study intervertebral discs because of its excellent soft-tissue contrast resolution and ability to differentiate disc sub-regions [4,26]. The most accepted grading classification for DDD on MRI images is the Pfirmann grading system, originally based on five grades based on disc structure, the distinction between NP and AF, signal intensity, and disc height in axial T2-weighted images (Figure 1) [27].

However, due to the low discriminatory power in assessing more advanced degenerative changes in the elderly spine, a modified Pfirrmann grading composed of eight grades was subsequently proposed [28].

The mainstay sequences are axial T2-weighted images, sagittal T1-weighted spin-echo, and T2-weighted spin-echo images. Additional sequences can be included on clinical needs, institution, and hardware, such as axial T1-weighted sequences, sagittal fat-suppressed T2-weighted sequences, and gadolinium-based contrast-enhanced T1-weighted sequences (especially in case of tumors, infections, and in the post-operative spine). In T2-weighted images, healthy IVDs display a hyperintense signal from the NP and the inner region of the AF, while the outer region of the AF is typically hypointense. In adults, it is common to see an intranuclear cleft of a horizontal dark signal, which is considered non-pathological [29].

With the development of DDD, a progressive reduction in the T2-weighted signal of the NP with loss of disc height occurs. In later stages, it is common to encounter a disc bulge or herniation, which are signs of facet osteoarthritis, and hypertrophy of the ligamentum flavum. However, MRI is less sensitive to early and potentially reversible changes of the degenerative cascade [10]. Thus, scientific interest in novel MRI tools to capture subtle changes of IVDs is progressively increasing.

## 4. Novel MR Imaging Tools for Intervertebral Disc Degeneration

In the last decade, many quantitative MRI techniques (QMRI), such as T1ρ, T2 and T2 star (T2*) mapping, diffusion-weighted imaging, sodium imaging, gagCEST, dGEMRIC, magnetization transfer, and spectroscopy, have been emerging as noninvasive, accurate diagnostic tools to detect early stages of DDD (Table 1).

These functional MRI techniques can investigate early biochemical and architectural changes within the disc, preceding structural changes and functional impairment [11,30]. Therefore, QMRI holds the potential to engage the IVD degenerative process at the beginning and possibly lead patients to regenerative treatments before the need for invasive surgical interventions [4,10,31,32,33]. However, these techniques are not widely used routinely due to several factors, including limited availability, the difficulty of acquisition with clinically possible scan times, and the lack of standardization and validation. The main characteristics of the most important studies investigating these techniques are summarized in Table 2.

### 4.1. T1ρ and T2 Relaxation Mapping

**Figure 2:**
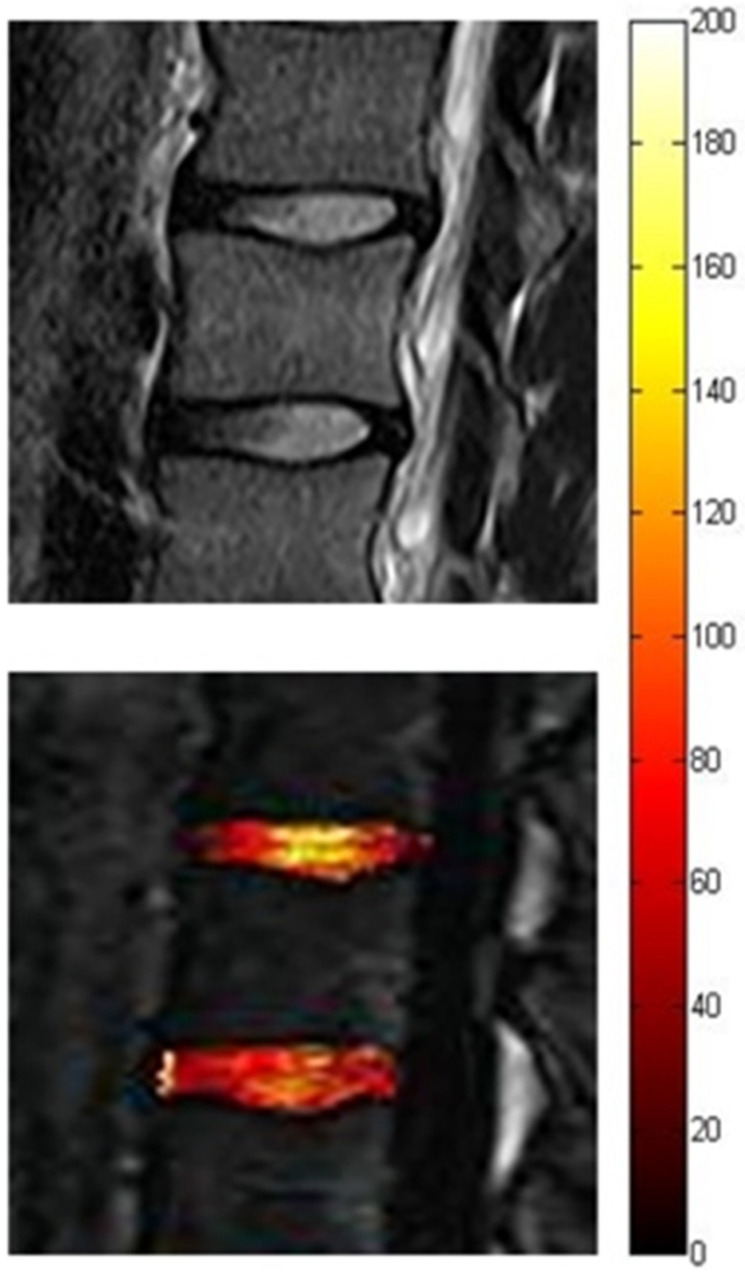
Color-coded map based on T1ρ images (lower panel) showing early disc degeneration (in red) at the levels L3–L4 relative to L2–L3, despite a similar signal intensity of the two discs in the T2-weighted image (upper panel).

T1ρ and T2 mapping are noninvasive MRI methods based on quantitative maps of relaxation time constants, reflecting local biochemical changes within the discs at early DDD before structural degeneration occurs [10]. Specifically, MRI T2 mapping relies on the T2 relaxation time to obtain maps that reflect mostly PG and water content and collagen anisotropy in IVDs. In contrast, MRI T1ρ mapping is based on the T1ρ relaxation time, which is obtained using spin-lock MR imaging. It is related to slow-motional interactions between macromolecules of the extracellular matrix and bulk water [32]. Compared with T2, T1ρ values show more sensitivity to hydration and PG content due to their greater dynamic range (Figure 2) [44].

In different stages of DDD, each disc region demonstrates other T2 and T1ρ relaxation values and, hence, variable signal intensity. Several studies showed that T1ρ and T2 values are higher in the NP due to its greater PG and water content than the AF, which is mainly composed of collagen fibers. Moreover, their values tend to decrease with aging and degeneration because of diminished water and GAG content within the disc [12,32]. Indeed, an inverse relationship between T2 and T1ρ relaxation values and Pfirrmann and Thompson grading systems were demonstrated [35,45,46,47]. T2 and T1ρ mapping have the potential to be used in the clinical routine since the pulse sequences and the software for generating quantitative maps are now available in commercial packages. These techniques do not require special preparation, contrast agent administration, or specific hardware. Moreover, T1ρ has a higher sensitivity in detecting PG reduction at early stages of cartilage degeneration compared with T2 mapping. However, it necessitates an additional RF pulse sequence, leading to a higher specific absorption rate (SAR). Novel technical developments are being studied to mitigate this risk [48].

### 4.2. Quantitative T2 Star (T2*) Mapping

**Figure 3:**
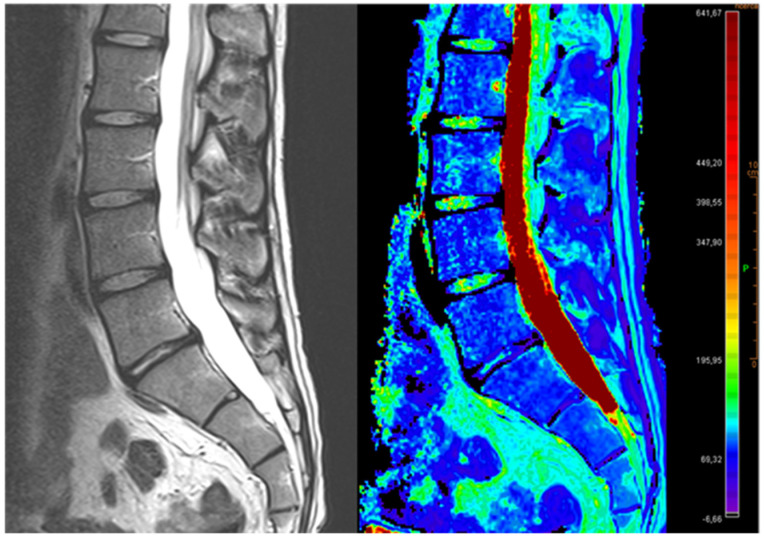
Illustration showing relative fluid content of IVDs. Representative pseudocolor images of an MRI T2-mapping analysis (**right**) and respective T2* mapping image (**left**).

T2* mapping exploits T2* relaxation times to evaluate the macromolecule architecture and water mobility in cartilage and IVDs (Figure 3) [49].

An inverse relationship between T2* values and DDD grading, such as Pfirrmann, has been demonstrated, particularly in patients with LBP. The main advantages of T2* over traditional T2 mapping are three-dimensionality, shorter acquisition time, and higher signal-to-noise ratio [50,51]. However, it is influenced by cartilage orientation and requires high field strengths and high RF pulse energy levels [52].

### 4.3. Diffusion-Weighted Imaging (DWI) and Diffusion Tensor Imaging (DTI)

DWI and DTI are non-invasive techniques that are able to study the IVD microstructural architecture. DWI and the relative ADC map measure water molecular diffusion, which reflects the composition and organization differences between tissues. DTI with fractional anisotropy (FA) maps identify water molecules’ motion direction inside IVDs. Usually, in IVDs, the NP has high ADC and low FA values because of the greater PG and water content. In contrast, the AF demonstrates low ADC and high FA values due to lower water content and collagen organization [12,34]. Several studies reported a progressive decrease in ADC and an increase in FA values, especially in the NP, associated with the onset and progression of DDD. Diffusion imaging is a promising marker for the integrity of the cartilage matrix; however, it is highly sensitive to motion artifacts and has a difficult balance regarding the SNR. Hence, further research is being conducted to improve the imaging resolution and possibly investigate IVD in a clinical setting on a routine basis [12,40,53,54].

### 4.4. Sodium Magnetic Resonance Imaging (^23^Na^+^_−_MRI)

Sodium concentration, which is detectable with sodium MRI, may be a feasible, noninvasive imaging indirect indicator of the GAG/PG content in the IVD and therefore of the pathophysiological alterations and temporal modifications related to DDD. This technique can quantify the ^23^Na^+^ ion content in tissues; cartilage and IVD, unlike most tissues in the human body, present a high level of such ions in the extracellular space [55]. Indeed, the NP extracellular matrix contains high concentrations of PG, which are formed by negatively charged GAGs that attract positively charged sodium ions (^23^Na^+^). PG depletion, which is typical of early DDD, determines a reduction of ^23^Na^+^, resulting in lower hydration, lower oncotic pressure, and collagen degradation [38]. Technical development of 3.0 T and 7.0 T systems provided an improvement of this technique, which is otherwise characterized by an inherently low signal-to-noise ratio (SNR) that is determined by lower sodium concentrations in tissues, lower gyromagnetic ratio, and shorter relaxation times than for proton (^1^H) imaging [55].

### 4.5. GAG Chemical Exchange Saturation Transfer (GagCEST)

Biochemical imaging with gagCEST directly quantifies GAG content, measuring the exchange of hydroxyl protons between GAG and bulk water [56]. Therefore, this technique may be used as a non-invasive tool to evaluate tissue composition and to depict pre-morphological IVD degeneration.

GAG depletion is thought to be a precursor of IVD degeneration, especially in the NP, as confirmed by research studies that reported lower gagCEST values in patients with chronic LBP compared with healthy individuals [10,56,57]. Even though gagCEST is a promising tool for the evaluation of early DDD, it is characterized by a reduced SNR and challenges in differentiating hydroxyl-GAG and water frequencies. Indeed, some studies demonstrated that 7 T MRI scanners could improve its performance and resolve these drawbacks [58].

### 4.6. Ultrashort TE (and Zero-TE Sequences)

Ultrashort echo time (UTE) is an MRI sequence based on TE < 1 ms that allows for acquiring a signal from tissues with short T2 components [59]. Since the CEP has a short T2 relaxation time, standard MRI sequences cannot depict its signal, while UTE may be exploited to assess its structure and thickness, especially in the early stages of DDD. CEPs appear with an intermediate/high signal with two layers, with one thick and superficial and the other thin and deep, corresponding to non-calcified and calcified cartilage, respectively [43,60]. Abnormal CEPs typically demonstrate signal loss, thinning, thickening, or irregularities in UTE MRI [61]. UTE is characterized by several shortcomings that should be overcome to achieve widespread clinical usage. Specifically, it does not hold an appropriate image contrast for the evaluation of long T2 tissues. Moreover, the low availability of UTE sequences in commercial platforms determines additional time and costs. UTE may be performed in addition to clinical sequences, increasing the overall scan time and costs.

### 4.7. Magnetic Resonance Spectroscopy

**Figure 4:**
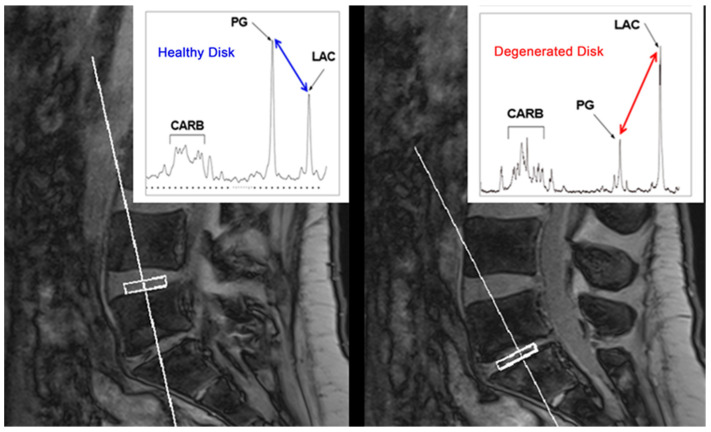
Illustrative MRS with a comparison of healthy and degenerated discs (courtesy of Aclarion, Inc., formerly Nocimed, Inc.). Please note the reduction of PG and the increase in LAC in the degenerated disc. CARB—carbohydrate/collagen, LAC—lactic acid, MRS—magnetic resonance spectroscopy, PG—proteoglycan.

Magnetic resonance spectroscopy (MRS) allows for analyzing the metabolic features of examined tissues by sampling the levels of metabolites based on regions of interest (ROIs; Figure 4) [31,59]. In DDD, the increase in biomarkers quantified using MRS (e.g., lactate, alanine, and lipid) indicates a state of hypoxia, inflammation, neovascularization, and neoinnervation; on the other hand, the GAG level, as a structural integrity marker, tends to decrease. In pathologic IVDs, anaerobic glycolysis increases with the augmented production of lactate and reduction of the pH, also determining the activation of metalloproteinases, which can break down the PG–collagen matrix. Therefore, MRS shows low GAG/collagen and GAG/lactate ratios but higher lactate/collagen ratios in pathologic IVDs, allowing for DDD identification and severity assessment [44]. MRS has already successfully been used in other anatomical sites, such as the brain; however, application in the musculoskeletal system is limited. It was applied for tumor characterization or muscle lipid quantification, though recent studies support its use to detect early and subtle biomarkers of DDD [31]. However, MRS measurements are affected by many factors, such as low signal-to-noise ratios, motion artifacts, long scanning times, and unavailability on standard MRI scanners. Hence, the feasibility and potential of this technique need to be further evaluated [44].

### 4.8. Delayed Gadolinium-Enhanced MRI of Cartilage (dGEMRIC)

dGEMRIC is an MRI technique using a gadolinium-based contrast agent that is capable of accelerating T1 relaxation times, making the tissues with gadolinium absorption brighter [10,62,63]. Since IVDs are avascular structures, oxygen and glucose are delivered via diffusion. Recent studies demonstrated that the dGEMRIC technique indirectly depicts the GAG content in the NP by measuring the diffusion rate, or rather, the negatively charged ions of a gadolinium-based contrast agent, which are inversely proportional to the GAG content itself [10]. The results in the literature are contradictory, with some studies demonstrating higher enhancement in degenerated discs, while others show a decreased T1 signal intensity after gadolinium injection [35]. The main drawback of dGEMRIC is the need for intravenous contrast administration, leading also to an increase in the scanning time of the procedure. The use of 7 T scanners and the omission of a pre-contrast T1 scan may be considered factors to decrease the scanning time [42,64].

### 4.9. Magnetization Transfer (MT) and MT Ratio (MTR)

The MT technique can characterize tissues by measuring chemical exchange processes between free and macromolecule-bound water protons [65]. The MT ratio (MTR) consists of an MT computation by acquiring two images, one with MT saturation and one without. MTR appears to be particularly sensitive to disc collagen content and the structural integrity of the matrix [3]. Research on the topic reported an increased MT in degenerated discs [3,65]. MT imaging is relatively easy to analyze, does not necessitate a specific coil, and is not characterized by long acquisition times. However, it seems to be less sensitive regarding quantitatively defining DDD compared with T2 mapping [41].

## 5. New Diagnostic Perspective: Artificial Intelligence

Artificial intelligence (AI) is quickly emerging in various branches of medicine [66]. Medical imaging analysis is currently one of the major fields of AI application in medicine. Concerning the spine, it was suggested that AI applied to MR images can yield a fast and accurate diagnosis and prognosis prediction regarding spinal diseases [67,68]. Disc localization; segmentation; and analysis of intensity, shape, and other features of IVDs in spine MRI are usually done by radiologists, with a subjective and time-consuming assessment that depends on individual experience and knowledge [69,70]. The use of AI to assess DDD in MR images has been investigated in several studies in the last decade. Indeed, it is of great interest to develop and test methods that automatically analyze discs using spinal MR images to quantify DDD objectively. For instance, it has been emphasized how AI with machine learning can obtain accurate and reliable grading of IVD degeneration in MRI scans [71]. We have recently performed a systematic review on the use of AI on computer vision applications regarding LBP. Studies on the topic exploited MRI technology to perform feature extraction and segmentation tasks, with accuracy rates and Sørensen–Dice coefficients > 85% in most studies [72].

Although these approaches are very reliable, user supervision is often required to validate and refine the results [73]. The analysis of IVD degeneration with AI in MR images will likely be further explored in the future since it can help radiologists facing work overload, assist clinical decision making, and reduce costs by improving indications for medical or surgical treatment in spinal disorders.

## 6. MRI as a Tool for Follow-Up Disc Regenerative Therapy

Conventional treatment in symptomatic DDD is usually conservative, including pharmaceutical therapy, such as analgesics and anti-inflammatory drugs, acting mainly on pain management, but also physical and behavioral therapies, such as structured exercise programs for rehabilitation and weight management, which are often underutilized. Surgical treatment is considered as a last resort in the case of end-stage degeneration and accompanying sequelae (e.g., disc herniation, spinal stenosis, and spondylolisthesis). However, these standard treatments do not directly target the degenerative mechanisms, leading inevitably to a progression toward severe disc disease [18,44].

Promising bioregenerative therapies for DDD are rapidly emerging, some of which are still in the early stages of preclinical development, such as intradiscal injection of growth factors, inflammatory or proteinase inhibitors, intracellular regulatory substances, gene or cell-based therapy, and tissue engineering with the development of biomaterials [74]. Gene therapy consists of the transfer of exogenous genetic material (DNA or RNA) into the genome of target cells to modulate gene expression by enhancing the synthesis of beneficial and/or missing proteins or by inhibiting the synthesis of detrimental products. Different from the administration of growth factors, by permanently modifying the host cell genome, this approach provides a long-term synthesis of the desired product. To date, multiple studies, both in vitro and in vivo, have demonstrated the feasibility and the beneficial potential of gene therapy for IDD. Several molecules and growth factors have been targeted, using either different viral vectors, non-viral vectors, or RNA interference, all yielding promising and significant results in terms of IVD structure preservation, increased matrix anabolism, and reduced cell loss [75].

However, the promising results of preclinical research have provided the basis for various clinical studies, which are substantially demonstrating the safety and efficacy of these techniques.

The purpose of these minimally invasive approaches is to modulate inflammation and restore the biochemical and histological composition of the disc by acting on matrix production, cell proliferation, and differentiation in order to decrease inflammation and pain, stimulate local repair and regeneration of damaged tissues, and overall, to slow down or even arrest the degenerative process. Patient selection is essential since end-stage degenerative disc disease does not benefit from regenerative therapies. The most suitable candidates for these therapies are subjects with early- or moderate-stage DDD with a high probability of disease progression [76].

Since between treatment and diagnosis, a mutual reliance subsists, it is fundamental to adopt an integrated approach in which the development of new therapies is accompanied by novel diagnostic tools for an early and personalized diagnosis. With the optimization of experimental strategies for the treatment of disc degeneration, new advanced diagnostic techniques are required.

Previous clinical studies investigating the role of intradiscal mesenchymal stem cell injection applied standard MRI sequences on T2-weighted images as the preferred imaging modality, both at baseline and as a follow-up, mostly evaluating water content, disc height, or Pfirmann grade [76,77,78]. Orozco et al. [79] reported a significant increase in water content in treated discs in T2-weighted sagittal images at 12 months following intradiscal cell transplantation. Similarly, Pettine and coauthors [80,81] showed an improvement of at least one Pfirrmann grade in 5 out of 10 one-level patients and 3 out of 10 two-level patients 12 months after the treatment. Such changes were positively associated with younger age and a higher transplanted cell concentration. Furthermore, all remaining patients maintained their previous Pfirrmann grade and did not worsen over the study period. In the study of Kumar et al. [82], the Pfirrmann grade of the transplanted disc decreased from grade IV to grade III at the 6-month and final follow-ups in one patient, while six patients showed an increase in water content based on the ADC mapping from DWI at the 12-month follow-up. In the first randomized controlled trial on intradiscal cell transplantation, Noriega et al. [83] did not find significant changes in terms of water content between the control and experimental groups. Nonetheless, in the controls, there was a deterioration from Pfirrmann grade 3.15 ± 0.15 to grade 3.78 ± 0.16 (*p* < 0.001), whilst in the cell-treated patients, there was an improvement from grade 3.68 ± 0.13 to 3.18 ± 0.17 (*p* < 0.01). In the recently published study by Amirdelfan and colleagues [84], no significant improvement in the modified Pfirmann grade in any group was noted at 6 months.

However, the literature is still sparse regarding the use of advanced MRI sequences of disc composition after bioregenerative treatments. Due to their ability to identify biochemical changes in disc composition, novel MRI sequences could be useful not only to detect early changes in disc composition before substantial morphological changes occur when is still possible to intervene in the progression of the disease but also for the evaluation of the regeneration process triggered after regenerative treatments [3,10,31].

Bioregenerative therapeutic interventions are promising techniques for the management of the early stages of DDD. In this context, novel compositional MRI sequences could be crucial, for patient selection, but also to monitor outcomes, assess the efficacy, and develop innovative regenerative strategies [3,31].

## 7. Conclusions

While standard MRI is the mainstay for evaluating intervertebral discs, many QMRI techniques are emerging, enabling non-invasive molecular characterization and microstructure evaluation of tissues. These techniques can recognize and measure early biochemical disc changes in DDD. In conclusion, despite their great potential, further technological improvements are needed to enable QMRI usage in clinical settings, allowing for early analysis and detection of IVDs degeneration and to evaluate the results of regenerative strategies.

## Figures and Tables

**Table 1:** Summary table of novel MRI techniques reported in normal and degenerated discs.

Technique	Biochemical Changes Evaluated	Normal IVD Intensity	Degenerated IVD Signal Intensity
T1ρ relaxation mapping	PG and water count, collagen anisotropy	High	Low
T2 relaxation mapping	PG and water content	High	Low
Quantitative T2* mapping	Macromolecule architecture and water mobility	High	Low
DWI with ADC and DTI with FA	Water diffusion, tissue composition and organization	High ADC Low FA	Low ADC High FA
^23^Na-MRI	Na^+^ concentration, GAG/PG content indirectly	High	Low
GagCEST	Exchange of hydroxyl-protons between GAG and bulk water, GAG content	High	Low
Ultrashort TE (and zero-TE sequences)	Tissue composition and organization	Intermediate/high High GAG/collagen	Low Low GAG/collagen
MRS	Levels of metabolites: lactate, alanine, GAG	High GAG/collagen High GAG/lactate Low lactate/collagen ratio	Low GAG/collagen Low GAG/lactate High lactate/collagen ratio
dGEMRIC	Diffusion rate, GAG content indirectly	High or low	Low or high
MT and MTR	Exchange process between free and macromolecule-bound water protons, collagen content and structural integrity of the matrix	MT high	MT high

^23^Na-MRI—sodium magnetic resonance imaging; ADC—apparent diffusion coefficient; DTI—diffusion tensor imaging; dGEMRIC—delayed gadolinium-enhanced MRI of cartilage; DWI—diffusion-weighted imaging; FA—fractional anisotropy; GAG—glycosaminoglycan; gagCEST—GAG chemical exchange saturation transfer; MRS—magnetic resonance spectroscopy; MT—magnetization; MTR—MT ratio; PG—proteoglycan; TE—time to echo.

**Table 2:** Main characteristics of representative studies investigating novel MRI techniques for DDD.

Authors	Country	Aim/Rationale	No. of Patients	MRI	Sequence	Main Conclusion
Perri et al. [34]	Italy	Evaluate the adequacy of DTI/FA mapping and T2-WI in the assessment of anisotropic water diffusion variations of AF fibers	75	3 T scanner	T2-WI FA/DTI	DTI and FA mapping can be useful in detecting AF fissures and lumbar disc herniation
Auerbach et al. [35]	USA	Assess the feasibility of T1ρ imaging to detect DDD	10	1.5 T scanner	T2-WI T1ρ-WI	T1ρ can be used as a non-invasive biomarker of proteoglycan loss and early DDD
Gornet et al. [36]	USA	Determine MRS usefulness in quantifying DDD severity and predict surgical outcomes	139	3 T and 1.5 T scanners	MRS	MRS correlates with Pfirrmann grade
Frenken et al. [37]	Germany	Evaluate gagCEST ability to detect GAG content in patients with LBP and lumbar radiculopathy	18	3 T scanner	GagCEST	GagCEST imaging is useful in detecting pre-morphological DDD
Vadapalli et al. [12]	India	Assess FA maps and T2 values ability to predict DDD	118	3 T scanner	T2-WI FA/DTI	FA maps and T2 values are potential biomarkers of DDD and predict disc health
Noebauer-Huhmann et al. [38]	Austria	Compare 7 T ^23^Na-MRI with T2 mapping and morphologic scoring at 3 T in the evaluation of lumbar IVDs	10	7 T and 3 T scanners	T2-WI ^23^Na-MRI	^23^Na-MRI and T2 mapping can help characterize biochemical changes in IVDs and are related to the Pfirrmann score
Yoon et al. [32]	South Korea	Assess T1ρ and T2 values correlation with Pfirrmann grades and morphologic changes	22	3 T scanner	T2-WI T1ρ-WI	T1ρ and T2 values present a correlation with DDD and morphologic changes in the IVD
Zobel et al. [39]	Italy	Evaluate T1ρ- and T2-WI for early degeneration assessment and correlate T1ρ value with Pfirrmann grade, sex, and BMI	63	1.5 T scanner	T2-WI T1ρ-WI	T1ρ values correlate with Pfirrmann grade and can be used to identify early DDD
Shen et al. [40]	China	Assess the capability of DWI, DTI, and T2* mapping to depict microstructural changes of early DDD	40	1.5 T scanner	ADC FA T2*-WI	ADC, FA, and T2* values may quantitatively reflect the microstructural characteristics of the NP
Wang et al. [41]	USA	Validate MTR as a noninvasive method for spatial quantification of IVD collagen content	4	1.5 T scanner	T2-WI MTR	MTR may serve as a noninvasive diagnostic tool for the diagnosis of early DDD
Schleich et al. [42]	Germany	Assess dGEMRIC feasibility as a biomarker for DDD	9	3 T scanner	dGEMRIC	Significantly lower dGEMRIC index suggested GAG depletion in DDD
Berg-Johansen et al. [43]	USA	Investigate the association between cartilage endplate thickness and DDD	6	3 T scanner	UTE T1ρ	UTE and T1ρ are associated with DDD

^23^Na-MRI—sodium magnetic resonance imaging; ADC—apparent diffusion coefficient; AF—annulus fibrosus; BMI—body mass index; DTI—diffusion tensor imaging; DDD—disc degenerative disease; dGEMRIC—delayed gadolinium-enhanced MRI of cartilage; DWI—diffusion-weighted imaging; FA—fractional anisotropy; GAG—glycosaminoglycan; gagCEST—GAG chemical exchange saturation transfer; IVD—intervertebral disc; LBP—low back pain; MRS—magnetic resonance spectroscopy; MTR—magnetization ratio; TE—time to echo; UTE—ultrashort echo time; WI—weighted imaging.

## Data Availability

The datasets used and/or analyzed during the current study are available from the corresponding author on reasonable request.
